# Identification of Novel Mutations in FAH Gene and Prenatal Diagnosis of Tyrosinemia in Indian Family

**DOI:** 10.1155/2012/428075

**Published:** 2012-10-30

**Authors:** Jayesh J. Sheth, Chitra M. Ankleshwaria, Rajeshwari Pawar, Frenny J. Sheth

**Affiliations:** ^1^Department of Molecular and Biochemical Genetics, Institute of Human Genetics, FRIGE House, Jodhpur Gam Road, Satellite, Ahmedabad, Gujarat 380 015, India; ^2^Department of Obstetrics and Gynecology, Jehangir Hospital, Sassoon Road, Pune 411 001, India

## Abstract

Carrier of tyrosinemia type I was diagnosed by sequencing *FAH* (fumarylacetoacetate hydrolase) gene. It leads to the identification of heterozygous status for both c.648C>G (p.Ile216Met) and c.1159G>A (p.Gly387Arg) mutations in exons 8 and 13, respectively, in the parents. The experimental program PolyPhen, SIFT, and MT predicts former missense point mutation as “benign” that creates a potential donor splice site and later one as “probably damaging” which disrupts secondary structure of protein.

## 1. Introduction

Hereditary tyrosinemia type-1 (HT1; 1 McKusick number 276700) is an autosomal recessive aminoacidopathy disorder affecting approximately one in 100,000 to 120,000 live births [1] with higher prevalence in the French, Canadian [2], and Scandinavian population [3]. Tyrosinemia type I results from the deficiency of the enzyme fumarylacetoacetate hydrolase (FAH) (EC 3.7.1.2) which is encoded by *FAH* gene. FAH is the terminal enzyme in the tyrosine catabolic pathway. In FAH deficiency, the immediate precursor, fumarylacetoacetate (FAA), is formed. The clinical spectrum of the disease is wide, ranging from chronic complications of hepatic failure to hepatocellular carcinoma, renal tubular dysfunction, renal failure, episodes of peripheral neuropathy, and death within the first few months of life. Etiology underlying these variables clinical outcome has not been elucidated and hence, less than 50% of the affected children are diagnosed when alive [4]. The accumulation of succinylacetone due to the deficiency of FAH enzyme can be detected in serum and urine. For the diagnosis of HT1, succinylacetone accumulation in the prenatal tissues like chorionic villi (CV) and amniotic fluid (AF) and mutation study of *FAH* gene could be carried out [5–9]. There has been one report from India confirming clinical diagnosis of HT1 on the basis of succinylacetone levels [10]. However, prenatal diagnosis based on sequencing of the *FAH* gene has not been reported. We hereby present, a nonconsanguineous family with index case confirmed as tyrosinemia type 1 by urine organic acid study and sequencing of *FAH* gene in the parents followed by prenatal diagnosis in subsequent pregnancy.

## 2. Case Report

**Figure 1:**
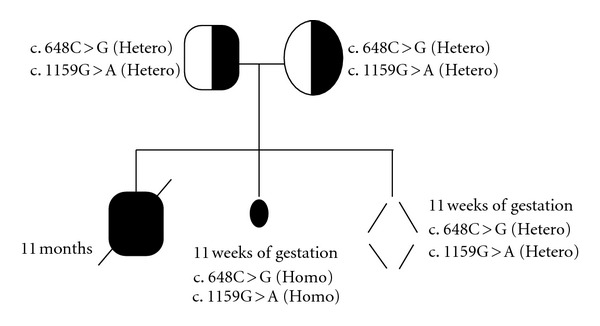
Pedigree.

**Figure 2:**
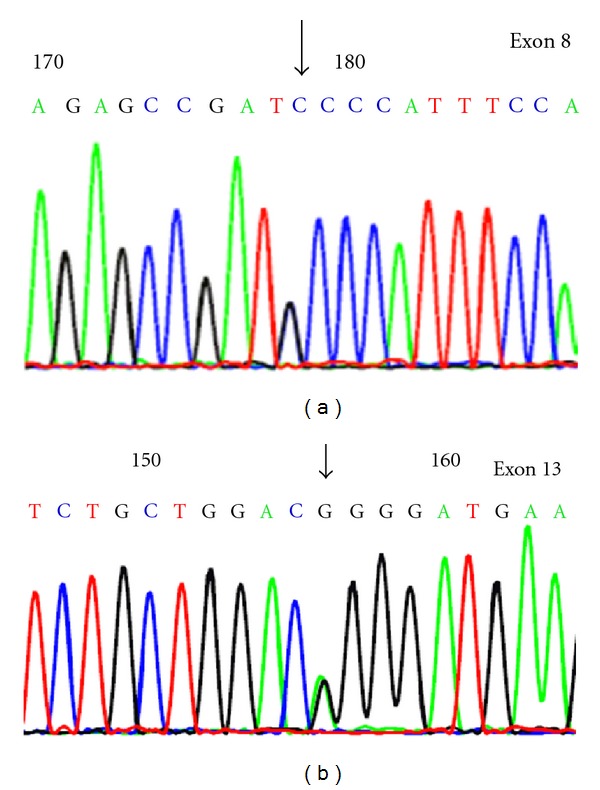
*FAH* gene sequencing study in heterozygous pregnancy (3rd gravida). (a) *FAH* gene sequencing study showing c.648C>G (p.Ile216Met) mutation in exon 8. (b) *FAH* gene sequencing study showing c.1159G>A (p.Gly387Arg) mutation in exon 13.

24-years-old woman with a nonconsanguineous marriage gave birth to a child who was diagnosed with failure to thrive, hepatosplenomegaly, and anemia at 4 months after birth. Ultrasonography of the liver showed portal hypertension, hepatomegaly with multiple hyper echoic nodules, dilated portal and splenic vein, and bilaterally enlarged kidneys. Biochemical analysis of the urine by gas chromatography showed more than 200-fold elevation of succinylacetone. At the age of 10 months, liver biopsy was carried out demonstrating postnecrotic mixed micro- and macronodular cirrhosis notably of metabolic origin. Serum alpha fetoprotein (AFP) level was >3000 ng/mL. Distinct biochemical abnormalities of increased succinylacetone concentration in the blood and urine, elevated plasma concentrations of tyrosine, methionine, phenylalanine, and elevated urinary concentration of tyrosine metabolites were observed in the child, which is consistent with the diagnosis of tyrosinemia type 1. This child died at the age of 11 months without confirmative molecular study of the *FAH* gene (Figure 1). The family approached one year later at 11 weeks of gestation for prenatal diagnosis and considering index case for biochemical diagnosis. Entire *FAH* gene sequencing was initially carried out in both parents, which leads to the identification of two distinct heterozygous point mutations. One in exon 8 is defined as c.648C>G, which is predicted to result in an amino acid substitution p.Ile216Met. Another heterozygous point mutation was detected in exon 13 as c.1159G>A, which is predicated to result in an amino acid substitution p.Gly387Arg. None of these mutations have been reported till date as shown in Table 1. Sequencing of *FAH *gene in the fetus was carried out and it showed homozygous mutations in both exon 8 and exon 13 for c.648C>G and c.1159G>A, respectively. Subsequently, the pregnancy was terminated. During the third pregnancy, prenatal study was carried out using CVS and AF demonstrated fetus with compound heterozygous status for both mutations as observed in the parents (Figure 2). Upon delivery, urine study for succinylacetone in the neonate was found to be normal confirming unaffected status of the child for HT1.

## 3. Discussion

HT1 results from the deficiency of FAH enzyme that catalyzes the final step in the tyrosine metabolic pathway. The precursor metabolite, fumarylacetoacetate, accumulates in the hepatocytes in the absence of FAH enzyme activity resulting in cellular damage as indicated in our case by raised serum AFP levels. This results in an increased excretion of tyrosine metabolites in the urine, especially succinylacetone, which was observed in the index case.

Other abnormalities such as elevated serum or plasma concentrations of tyrosine, methionine, and phenylalanine in the index case were consistent with the diagnosis of tyrosinemia type 1 [4]. During the subsequent pregnancy, confirmation of HT1 was carried out by sequencing of *FAH* gene in both parents. It confirmed heterozygous mutation for c.648C>G (p.Ile216Met) in exon 8 for both parents. The amino acid p.Ile216 residue is conserved amongst FAH proteins from human, mouse, rat, chicken, and frog. Experimental program PolyPhen2 (Polymorphism Phenotyping v2) predicted this amino acid substitution as “benign” with a score of 0.187. MT (MutationT@ster) and SIFT (Sorting Intolerant from Tolerant) has predicted this point mutation as nonpathogenic. Although, MT suggests that this mutation probably creates a novel splice site. Additionally, methionine is a sulphur containing amino acid, which may create *de novo* disulfide linkages within the protein. Both parents were also heterozygous for a mutation in exon 13 of *FAH* gene of unknown clinical significance identified as c.1159G>A (p.Gly387Arg). This residue is also conserved among FAH protein in human, primates, mouse, rat, chicken, and frog. The experimental program PolyPhen predicts this amino acid substitution as “probably damaging” with a score of 1.0 for disrupting the secondary structure of the protein [15]. Secondary structure of FAH protein (PDB ID 1QCO) contains 21 beta sheet turns. Turn number 20 between two beta sheets take place at the 387 amino acid position which is occupied by glycine, the smallest amino acid. When glycine is replaced with arginine, which is a larger electrically charged molecule, the turn number 20 might be disrupted conferring damaging properties of p.Gly387Arg. During the second pregnancy, prenatal diagnosis at 11 weeks of gestation identified both aforementioned homozygous mutations in the fetus and pregnancy was subsequently terminated. However, confirmative study in the abortus material was not carried out. During the third pregnancy, prenatal study was carried out from CVS and AF demonstrating compound heterozygous status for the above-mentioned mutations in the fetus, which is similar to that observed in the parents confirming carrier status for HT1. Subsequent biochemical analysis at birth confirmed the molecular study result.

Present study demonstrates a novel disease causing missense mutation in exon 13, which potentially disrupts the secondary structure of FAH protein. A novel missense point mutation that probably creates a donor splice site in the exon 8 of *FAH* gene for tyrosinemia type I has also been identified. In an Indian family these mutations have not been reported till date.

## Figures and Tables

**Table 1:** Frequency of mutations found in *FAH* gene all over the world*.

Population	Mutations	Frequency
Ashkenazi Jewish	p.Pro261Leu (P261L)	~100%
Finnish	p.Trp262X (W262X)	N/A
French Canadian	c.1062+5G>A (IVS 12+5G>A)	87.9%
Pakistani mutation	p.Gln64His (Q64H)	N/A
Scandinavian	p.Gly337Ser (G337S)	N/A
Turkish	p.Asp233Val (D233V)	N/A
Northern European	c.1062+5G>A (IVS 12+5G>A)	60%
Southern European	c.554-1G>T (IVS 6-1G>T)	N/A
Indian (Present study)	p.Ile216Met and p.Gly387Arg	N/A

*The above mentioned population-specific mutations result from founder effect or genetic drift [9, 11–14].

## References

[1] Mitchell GA, Grompe M, Lambert M, Tanguay RM, Scriver CR, Beaudet AL, Sly WS, Valle D (2001). Hypertyrosinemia. *The Metabolic and Molecular Bases of Inherited Disease*.

[2] De Braekeleer M, Larochelle J (1990). Genetic epidemiology of hereditary tyrosinemia in Quebec and Saguenay-Lac-St-Jean. *American Journal of Human Genetics*.

[3] Kvittingen EA (1986). Hereditary tyrosinemia type I—An overview. *Scandinavian Journal of Clinical and Laboratory Investigation*.

[4] King LS, Trahms C, Scott CR, Pagon RA, Bird TD, Dolan CR (2006). *Tyrosinemia Type 1 Gene Reviews*.

[5] Lindblad B, Lindstedt S, Steen G (1977). On the enzymic defects in hereditary tyrosinemia. *Proceedings of the National Academy of Sciences of the United States of America*.

[6] Melancon SB, Gagne R, Grenier A, Lescault A, Dallaire L, Laberge C, Fisher MM, Roy CC (1983). Deficiency of fumarylacetoacetase in the acute form of hereditary tyrosinemia with reference to prenatal diagnosis. *Pediatric Liver Disease*.

[7] Stoner E, Starkman H, Wellner D (1984). Biochemical studies of a patient with hereditary hepatorenal tyrosinemia: Evidence of glutathione deficiency. *Pediatric Research*.

[8] Gagne R, Lescault A, Grenier A (1982). Prenatal diagnosis of hereditary tyrosinaemia: measurement of succinylacetone in amniotic fluid. *Prenatal Diagnosis*.

[9] Elpeleg ON, Shaag A, Holme E (2002). Mutation analysis of the FAH gene in Israeli patients with tyrosinemia type I. *Human mutation*.

[10] Bijarnia S, Puri RD, Ruel J, Gray GF, Jenkinson L, Verma IC (2006). Tyrosinemia type I—diagnostic issues and prenatal diagnosis. *Indian Journal of Pediatrics*.

[11] Bergman AJ, van den Berg IE, Brink W, Poll-The BT, Ploos van Amstel JK, Berger R (1998). Spectrum of mutations in the fumarylacetoacetate hydrolase gene of tyrosinemia type 1 patients in northwestern Europe and Mediterranean countries. *Human Mutation*.

[12] Bergeron A, D’Astous M, Timm DE, Tanguay RM (2001). Structural and functional analysis of missense mutations in fumarylacetoacetate ydrolase, the gene deficient in hereditary tyrosinemia type 1. *Journal of Biological Chemistry*.

[13] Arranz JA, Piñol F, Kozak L (2002). Splicing mutations, mainly IVS6.1 (G>T), account for 70% of fumarylacetoacetate hydrolase (FAH) gene alterations, including 7 novel mutations, in a survey of 29 tyrosinemia type I patients. *Human Mutation*.

[14] Heath SK, Gray RGF, McKierman P, Au KM, Walker E, Green A (2002). Mutation screening for tyrosinaemia type 1. *Journal of Inherited Metabolic Disease*.

[15] Tchernitchko D, Goossens M, Wajcman H (2004). In silico prediction of the deleterious effect of a mutation: proceed with caution in clinical genetics. *Clinical Chemistry*.

